# Management of Milch Cows in the Fall and Winter

**Published:** 1855-03

**Authors:** 


### MANAGEMENT OF MILCH COWS IN THE FALL AND WINTER.

How a city mouth waters for a pitcher of rich pure milk from the farm-house. A correspondent of the Cincinnati Times, thus discourses :—

"In a former number you made the following remarks :— That beets, parsnips and carrots were excellent to produce milk ; but you say you prefer carrots to produce not only rich milk, but rich butter ; and you ask if there is a better vegetable that can be grown for milchcows, all things considered. Let us hear from those who are in favor of its cultivation. There are many kinds of feed used for cows, such as slops or swill, and malt from distilleries ; but this cannot be had by every one who keep cows for the production of milk. I hope the day is not far distant when such feed cannot be had for cows or any other animal.

Before the blight came on the potatoe, that tuber was more extensively grown to feed to cows in order to produce large quantities of milk, but not of good quality. I do not think it would do to raise potatoes to feed cows at present prices.

The vegetable I wish to recommend as the best, all things considered, is white, flat turnips. Some, perhaps, will object to the turnip, because it will affect the taste of milk and butter. So it does if fed raw; this can be avoided by boiling. For each cow, boil half a bushel of turnips soft; while hot add five or six quarts of shorts, which will swell, and you will get the worth of it. A mess like this fed to a cow one day, will produce more milk of a good quality than any other feed of the same cost. Turnips fed in this way, do not taint the butter or milk. One thing in favor of turnips as food for cows is, they can be sown as late as August, or the first of September. I sowed some as late as September last year, which were very fine. Turnips are also very profitable for pigs, when boiled in the same way as for cows.

